# Contribution of Pannexin1 to Experimental Autoimmune Encephalomyelitis

**DOI:** 10.1371/journal.pone.0066657

**Published:** 2013-06-20

**Authors:** Sarah E. Lutz, Estibaliz González-Fernández, Juan Carlos Chara Ventura, Alberto Pérez-Samartín, Leonid Tarassishin, Hiromitsu Negoro, Naman K. Patel, Sylvia O. Suadicani, Sunhee C. Lee, Carlos Matute, Eliana Scemes

**Affiliations:** 1 Dominick P. Purpura Department of Neuroscience, Albert Einstein College of Medicine, Bronx, New York, United States of America; 2 Departamento de Neurociencias, Universidad del País Vasco, Leioa, Spain; 3 Department of Pathology, Albert Einstein College of Medicine, Bronx, New York United States of America; 4 Department of Urology, Albert Einstein College of Medicine, Bronx, New York, United States of America; Julius-Maximilians-Universität Würzburg, Germany

## Abstract

Pannexin1 (Panx1) is a plasma membrane channel permeable to relatively large molecules, such as ATP. In the central nervous system (CNS) Panx1 is found in neurons and glia and in the immune system in macrophages and T-cells. We tested the hypothesis that Panx1-mediated ATP release contributes to expression of Experimental Autoimmune Encephalomyelitis (EAE), an animal model for multiple sclerosis, using wild-type (WT) and Panx1 knockout (KO) mice. Panx1 KO mice displayed a delayed onset of clinical signs of EAE and decreased mortality compared to WT mice, but developed as severe symptoms as the surviving WT mice. Spinal cord inflammatory lesions were also reduced in Panx1 KO EAE mice during acute disease. Additionally, pharmacologic inhibition of Panx1 channels with mefloquine (MFQ) reduced severity of acute and chronic EAE when administered before or after onset of clinical signs. ATP release and YoPro uptake were significantly increased in WT mice with EAE as compared to WT non-EAE and reduced in tissues of EAE Panx1 KO mice. Interestingly, we found that the P2X7 receptor was upregulated in the chronic phase of EAE in both WT and Panx1 KO spinal cords. Such increase in receptor expression is likely to counterbalance the decrease in ATP release recorded from Panx1 KO mice and thus contribute to the development of EAE symptoms in these mice. The present study shows that a Panx1 dependent mechanism (ATP release and/or inflammasome activation) contributes to disease progression, and that inhibition of Panx1 using pharmacology or gene disruption delays and attenuates clinical signs of EAE.

## Introduction

In multiple sclerosis (MS) and in the animal model experimental autoimmune encephalomyelitis (EAE), the acute disease state is associated with T cell extravasation into the CNS, elevated levels of macrophage/monocyte derived cytokines such as interleukin-(IL)-1β, and macrophage mediated myelin phagocytosis, whereas the chronic disease phase is associated with ongoing cellular losses [1], [2].

ATP is involved in diverse signaling platforms and is well known for inducing excitotoxic cell death within the nervous system. In myelinated tissue, ATP initiates an excitotoxic cascade that culminates in apoptosis of oligodendrocytes. Pharmacologic inhibition of the ATP-sensitive ionotropic P2X_7_ receptor (P2X_7_R) reduces demyelination, axonal damage, and oligodendrocyte cell death in white matter ischemia and in EAE [3], [4]. The non-lytic mechanism of ATP release in EAE has yet to be defined.

Under whole-cell patch clamp recordings, P2X_7_R activation by ATP is marked by an initial small conductance followed by a protracted larger conductance permissive to molecules up to ∼1 kDa that is due to recruitment of Pannexin1 (Panx1) channels [5]–[7]. Because of their association, P2X_7_R activation can trigger cellular ATP release by opening Panx1 channels, and thereby the P2X_7_R-Panx1 complex can function as an ATP-sensitive ATP release unit. Independently of P2X_7_R, Panx1 channels can also be activated by voltage, mechanical stretch, and extracellular K^+^, with high [K^+^]_out_ activating Panx1 channels in neurons and astrocytes [8]–[10]. Panx1 channels can be blocked by gap junction channel blockers but at much lower concentrations [11], including mefloquine (MFQ), a quinine derivative that reversibly blocks Panx1 channels at the nM range [12].

Expression of Panx1 in neurons and astrocytes indicates that these channels could be involved in neurodegeneration. Indeed, Panx1-mediated ATP release was documented for cultured spinal cord astrocytes and oligodendrocytes in response to inflammatory mediators and to oxygen/glucose deprivation [3], [13]. In *vivo*, Panx1 has been shown to contribute to neuronal cell death in retinal ischemia [14] and in enteric colitis [15].

In the present study, we tested the hypothesis that inhibition or deletion of Panx1 improves neurologic and pathologic signs of EAE. We show that enhanced Panx1 activity and ATP release is characteristic of the EAE spinal cord. Moreover, blockade of Panx1 channels with MFQ or genetic deletion of Panx1 confers some degree of protection against clinical and pathological signs of EAE, which occurs in part by reducing the infiltration of inflammatory cells during the acute phase and by attenuating the amount of ATP release from diseased CNS tissue. Nevertheless, during the chronic phase of the disease, when no improvement of clinical signs was conferred by deleting Panx1, the upregulation of P2X_7_R found in EAE spinal cords most likely overcomes the decreased ATP release seen in Panx1 KO.

## Materials and Methods

### Ethics Statement

Mice were housed and maintained under specific pathogen-free conditions in the Animal Resource Facilities of the University of Basque Country and the Albert Einstein College of Medicine and all experiments were pre-approved by the respective Institutional Animal Care and Use Committees (IACUC approval numbers AUP/2010-0413; CEBA/261/2012 and CEBA/136/2011).

### Animals

Male adult Lewis rats and female C57Bl/6 mice were obtained from IFFA CREDO and Charles River, respectively. Panx1 deficient mice were obtained from UCDavis KOMP (allele: Panx1^tm1a(KOMP)Wtsi^) as heterozygous (HT) mice on C57Bl/6 background and bred to homozygosity (Panx1 KO and Panx1 WT) and maintained in an SPF animal facility at Albert Einstein College of Medicine. Only female mice were used in this study.

### Induction of Experimental Autoimmune Encephalomyelitis (EAE)

Acute EAE was induced in Lewis rats with inoculum containing 100 µg of guinea pig myelin basic protein (MBP; Sigma) in water, emulsified in equal volumes of Incomplete Freund’s Adjuvant (Sigma), supplemented with 500 µg of heat-inactivated *M. tuberculosis* H37Ra (DIFCO Laboratories). Chronic EAE was induced in 8–10 week old female mice by subcutaneous immunization with 300 µg of myelin oligodendrocyte glycoprotein MOG_35–55_ peptide (MEVGWYRSPFSRVVHLYRNGK; Celtek Bioscience) in a 200 µl emulsion composed of equal parts MOG (in dH_2_O) and Incomplete Freund's Adjuvant (BD Biosciences) supplemented with heat-killed *Mycobacterium tuberculosis* H37Ra (BD Biosciences) at 10 mg/mL. The day of MOG immunization was designated day 0. On day 0 and day 2 post-immunization (dpi), mice were injected intraperitoneally with 500 ng Pertussis toxin (List Biological Laboratory). Clinical signs of disease were scored in a 0–8 scale where, 0: No signs; 1: Loss of tail tone; 2: Paralyzed tail; 3: Hindlimb weakness; 4: Hindlimb hemiparalysis; 5: Complete hindlimb paralysis; 6: Complete hindlimb paralysis with forelimb weakness; 7: Tetraplegia; 8: Moribund.

### Mefloquine Treatment

Daily i.p. injections of the Panx1 channel blocker mefloquine (MFQ; Bioblocks-QU024-1) were started 14 days post-immunization of female mice and clinical signs followed for 38 days. In some experiments, rats and mice received daily i.p. injections of MFQ starting at one week post-immunization and followed thereafter for one or two weeks.

### Axon Conduction Latency

Conduction latency of axons of the corticospinal tract of EAE mice treated and untreated with MFQ was measured as previously described [4]. Briefly, conduction latency was measured in anesthetized mice (tribromoethanol: 240 mg/kg, i.p.; Sigma-Aldrich) using a silver chloride recording electrode placed in the vertebral canal at the L2 level and a stimulatory (0–90 V, 0.2 Hz, 50 µs) bipolar electrode placed on the surface of the primary motor cortex (1 mm anterior from Bregma, 2 mm from midline).

### Dye Uptake

Mouse spinal cords were removed by insufflation with cold PBS, cut into 330 µm slices using a tissue chopper and immediately incubated in air-bubbled ACSF (145 mM NaCl, 2.5 mM KCl, 3.1 mM CaCl_2_, 1.3 mM MgCl_2_, 10 mM glucose, 10 mM HEPES, pH 7.4) for 30–40 min, at 34°C. Uptake of the dye YoPro was performed as previously described [9]. For that, cervical and thoracic spinal cord slices from non-EAE and EAE mice were bathed for 1 h in ACSF containing 5 µM YoPro-1 (Molecular Probes; Invitrogen), washed 3×10 min with ACSF, and fixed in 4% paraformaldehyde overnight. YoPro fluorescence was measured using an epifluorescence microscope equipped with a 4x objective, 488/512 nm excitation/emission filter sets and MetaFluor software version 7.1 (Molecular Devices). YoPro fluorescence was measured from 15–25 slices of cervical/thoracic spinal cord from each mouse. Fluorescence intensity values obtained from regions of interested placed on images obtained from the gray matter tissues of EAE mice were normalized to non-EAE controls and expressed as mean ± s.e.m.

### Quantification of Cell Death

Cell death was assessed by measuring propidium iodide (PI) fluorescence from spinal cord slices incubated for 10 min in ACSF containing PI (10 µM) prior to the addition of YoPro. Spinal cords slices were processed as described above and PI fluorescence intensity was measured from regions of interest placed in the gray matter, using 594 nm excitation filter set.

### ATP Assays

ATP released into the ACSF bathing lumbar and sacral spinal cord slices was measured with a Promega Luciferin/Luciferase assay kit and a Turner luminometer. Fifty microliters of ACSF were collected after 30 min incubation and the amount of ATP present in the ACSF normalized to total amount of protein.

### Protein Measurements

Spinal cord slices were sonicated in lysis buffer (150 mM NaCl, 10 mM Tris-base, 1% Triton-X, pH 7.4) containing protease inhibitor cocktail (complete, EDTA-free; Roche). After spin down supernatants were used to measure protein content using the BCA assay (Thermo Scientific).

### Splenocyte Cultures

Spleens from Panx1 WT and KO mice were homogenized with a 1 ml syringe plunger, passed through a 40 µm filter, centrifuged 8 min at 250 g, and re-suspended in red blood cell lysis buffer (155 mM NH_4_Cl, 10 mM KHCO_3_, 0.1 mM EDTA, pH 7.2) for 5 min with occasional shaking. Cells were centrifuged and resuspended in Dulbecco’s Modified Eagle Medium (DMEM; Gibco) supplemented with 10% fetal bovine serum (FBS; Gibco), 1% penicillin/streptomycin (P/S; Gibco), and MEM non-essential amino acids (Gibco). Cells were plated at a density of 6×10^7^ per 35 mm dish. The next day, non-adherent cells were removed by shaking. Adherent cells were maintained in culture for 2 weeks before use, at which time we found by immunocytochemistry that about 97% cells expressed the macrophage marker CD11b.

### IL-1β ELISA Assay

Panx1 WT and KO splenic macrophages were incubated in serum-free DMEM, 1% P/S, MEM non-essential amino acids overnight, and then treated with 1 µg/ml lipopolysaccharide (LPS; Sigma-Aldrich) in media overnight. Cells were then washed in phosphate buffered saline (Dulbecco’s PBS) and exposed for 20 min to 5 mM ATP (Sigma). Supernatants were collected at the end of the 20 min ATP stimulation, centrifuged for 10 sec at 10,000 rpm to remove cellular debris, and the clarified supernatant was used for mouse IL-1β ELISA according to manufacturer instructions (PeproTech). The amount of IL-1β in the supernatant was normalized to the total cellular protein levels obtained from cells harvested in 100 µl lysis buffer (1% TritonX-100, 150 mM NaCl, 10 mM Tris-base, Roche EDTA-free complete protease inhibitor; pH 7.4) using the BCA assay. Data are reported as mean ± SEM of triplicate measurements of IL-1β. Three independent experiments were performed.

### Histopathology

Non-EAE and EAE mice were anesthetized with isoflurane, perfused with 20 ml PBS and 20 ml 4% paraformaldehyde, and spinal cords dissected. In some experiments, spinal cords were removed by insufflation with cold PBS. Paraffin-embedded sections from thoracic, lumbar and sacral spinal cord were stained with hematoxylin and eosin (H & E). Lesions were quantified in the ventral funiculus. Areas of parenchymal inflammation were manually outlined, measured using NIH ImageJ software [16] and presented as a percentage of ventral funiculus white matter area.

### Western blotting

Mice were anesthetized with isoflurane, decapitated, and spinal cords removed by insufflation with cold PBS. Lumbar spinal cord was sonicated in lysis buffer containing protease inhibitors. Each sample (30 µg) was electrophoresed on 4–20% gradient sodium dodecyl sulfate-polyacrylamide minigels (BioRad) and transferred to nitrocellulose membranes. Membranes were blocked with 2% milk and incubated for 2 hours with antibodies to P2X_7_R (Alomone Lab., 1∶1000) or GAPDH (Fitzgerald, 1∶10,000). HRP-conjugated secondary antibodies were used at 1∶3000 (Santa Cruz). Membranes were exposed to enhanced chemilluminescent substrate (Millipore) and detected with X-ray films. Densitometric analysis of bands was performed with ImageJ.

### qRT-PCR

Cerebellar tissues of 3 naïve and 3 EAE wild-type mice were used to quantify the levels of *Panx1* transcripts. Tissues were minced and homogenized with a Bullet Blender (Next Advance Inc.) and total RNA was extracted using the plus mini kit (Qiagen) according to the manufacturer’s protocol. Complementary DNA was synthesized from 1 µg/10 µl of RNA, using a Superscript VILO cDNA Synthesis Kit (Invitrogen). Primers used were Pannexin1 (F: AGCCAGAGAGTGGAGTTCAAAGA; R: CATTAGCAGGACGGATTCAGAA) and 18S ribosomal RNA (F: CACGGCCGGTACAGTGAAAC; R: AGAGGAGCGAGCGACCAAA). The ribosomal *18S* was used as a house-keeping gene for normalization. Real-time qRT–PCR was performed using SYBR Green PCR Master Mix with 7300 Fast Real-Time PCR system (Applied Biosystems). Reaction mixtures were denatured at 95°C for 10 min, followed by 40 PCR cycles. Each cycle consisted of the following three steps: 94°C for 15 sec, 57°C for 15 sec, and 72°C for 1 min. Each sample was normalized against internal control (*18S* ribosomal RNA); the relative values for target abundance was extrapolated from standard curves generated from the reference standard.

### Statistical Analyses

Statistical comparisons were made using GraphPad Prism 5.0 software. Clinical signs of disease were compared between groups using unpaired student’s t-test or one-way ANOVA followed by Newman-Keuls post hoc analysis, as indicated in the text. *P*<0.05 was considered significant.

## Results

### Blockade of Panx1 Channels Delays Onset and Ameliorates EAE Signs

**Figure 1:**
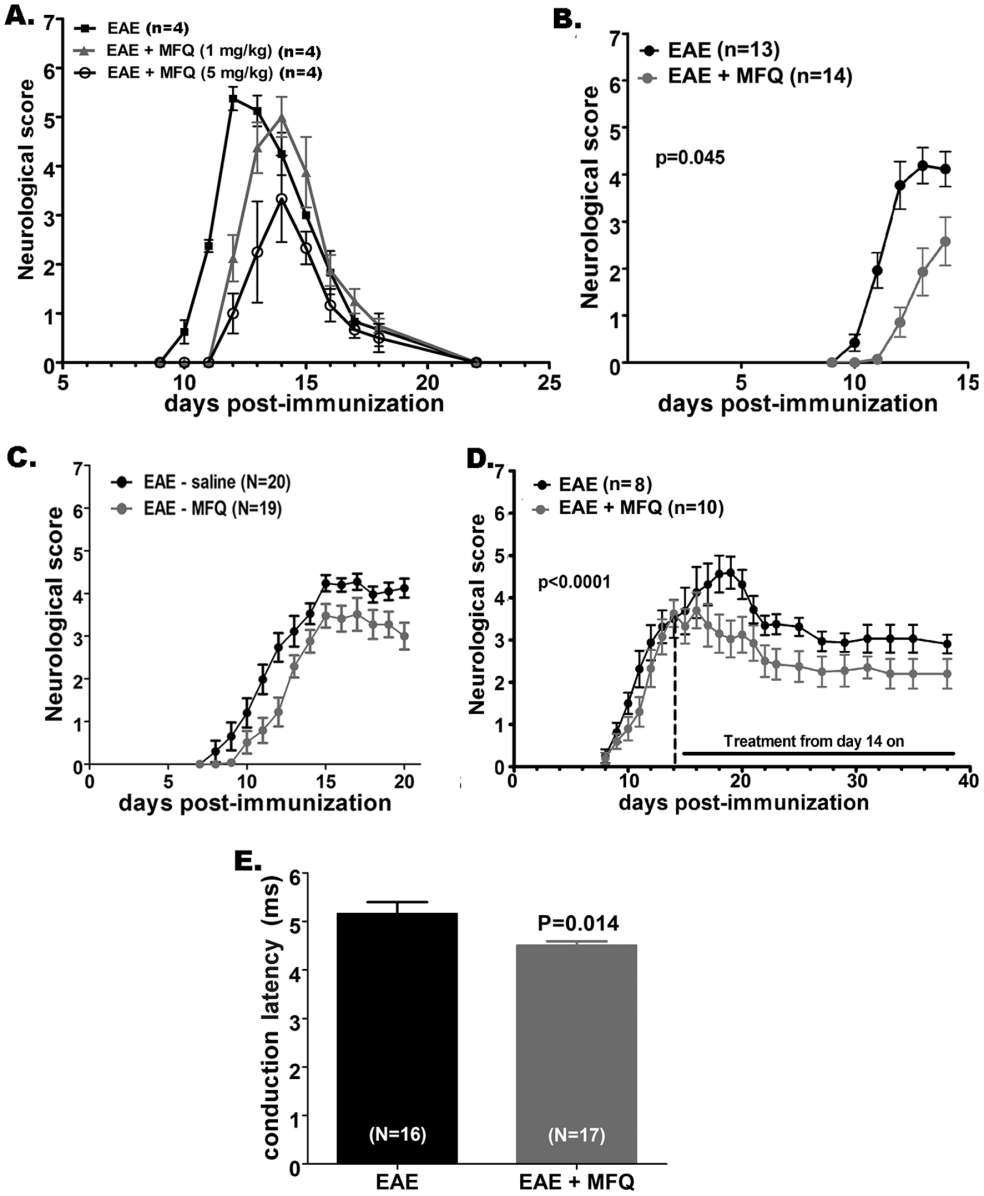
Blockade of Panx1 channels ameliorates signs of EAE. Time course of the mean ± s.e.m. values of neurological scores recorded from rats (**A, B**) and mice (**C, D**) with EAE. (**A**) Daily intraperitoneal injections of 5 mg/kg mefloquine (MFQ) but not 1 mg/kg MFQ improved EAE outcome in rats. (**B–D**) Daily injections of MFQ (5 mg/kg) administered to (**B**) rats and (**C**) mice at 7 days post immunization (dpi) till, respectively 14 and 20 dpi, and to (**D**) mice starting at 14 dpi till 38 dpi is shown to ameliorate the EAE symptoms. P values were calculated from all data points using Mann-Whitney test. (**E**) Bar histograms showing the mean ± s.e.m. values of conduction latency in the corticospinal pathway of mice with EAE untreated (black bar) and treated (gray bar) with MFQ. In parentheses are the numbers of animals used. P values were obtained from unpaired t-test.

The contribution of Panx1 channels to EAE was first evaluated using the transient EAE model in rats immunized with MBP that were either untreated or treated daily with MFQ (1.0 and 5.0 mg/kg; starting 7 days post-immunization: 7 dpi). As shown in Fig. 1A, a significant improvement in neurological scores was measured in rats that received the highest concentration of the Panx1 channel blocker (P = 0.022, paired t-test; N = 4 animals per group). When administered at 5 mg/kg, MFQ delayed the disease onset and reduced the EAE symptoms compared to those of untreated rats (Fig. 1B; P<0.05; N = 13–14 rats). Similarly to what was recorded in rats, daily injections of MFQ (5.0 mg/kg; starting 7 dpi) in mice immunized with MOG also delayed onset and reduced EAE symptoms compared to MFQ-untreated EAE mice (Fig. 1C; P<0.05; N = 19–20 mice).

In order to evaluate whether the beneficial effect of MFQ was related to an attenuation of peripheral and/or CNS inflammatory responses, we injected MFQ (5 mg/kg) in mice after the onset of the disease. Fourteen days post-immunization, a time point in which the majority of mice had a score 4 (hindlimb hemi-paralysis), MFQ was injected daily and scoring performed for 24 more days. As indicated in Fig. 1D, a slight but significant improvement of symptoms was recorded in female mice that received the Panx1 channel blocker, with MFQ-treated mice displaying on average at day 38 post-immunization paralyzed tail and the untreated ones hindlimb weakness.

Further evidence for the beneficial effect of MFQ was obtained by measuring the conduction latency in spinal tracts of EAE mice treated and untreated with MFQ (5 mg/kg). Untreated EAE mice displayed a conduction latency of 5.2±0.24 ms (N = 16 mice) while EAE mice that were daily treated with MFQ had a significantly shorter conduction latency (4.5±0.08 ms; N = 17 mice; P<0.05; Fig. 1E).

Thus, together these data indicate a beneficial effect of MFQ and suggest that Panx1 channels contributed to the disease, both by delaying the onset and attenuating EAE symptoms.

### Delayed Onset and Reduced Mortality in Panx1 KO Mice with EAE

**Figure 2:**
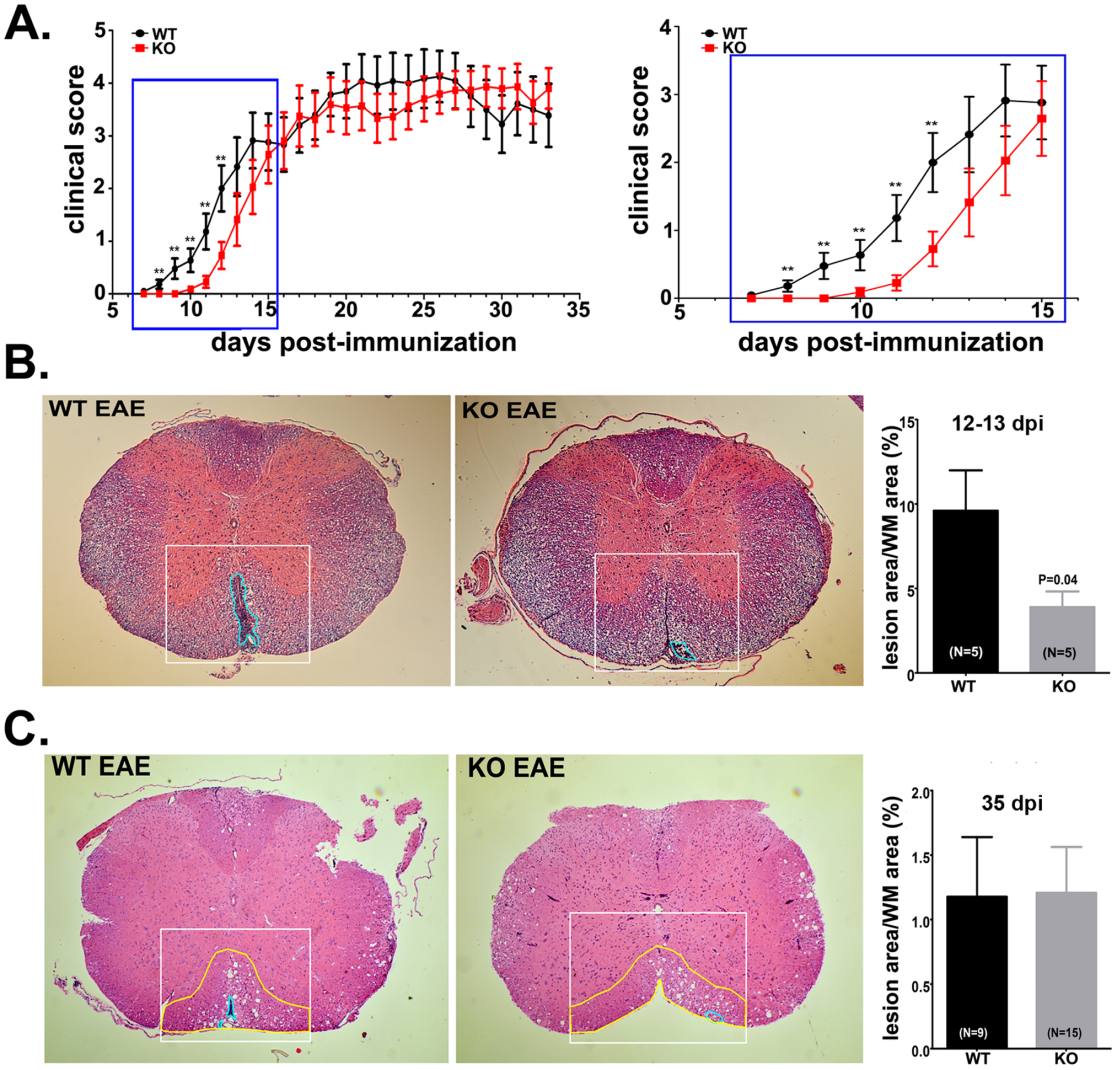
Delayed onset of EAE in Panx1 KO mice. (**A - right**) Time course of clinical signs recorded from Panx1 wild type (WT) and Panx1 knockout (KO) mice immunized for MOG. (**A-left**) The graph depicts the initial acute phase of the disease (blue rectangle in **A**) showing that up to day 12 post-immunization, a significant difference in clinical scores was detected between the two genotypes. Symbols represent mean ± s.e.m. Twenty two Panx1 WT and 22 Panx1 KO female mice were immunized. Thirteen days post-immunization (dpi), 5 animals of each genotype were used for histopathology; 6 Panx1 WT and 2 Panx1 KO died or were euthanized due to the severity of EAE symptoms. *P<0.01, unpaired t-test. (**B**) Hematoxylin & eosin stained sections of spinal cord from Panx1 WT and Panx1 KO mice obtained at the acute phase of EAE (12–13 dpi). Ventral funiculus is outlined in white box and inflammatory lesions are outlined in cyan. Bar histograms on the right represent the mean ± s.e.m values of the percent lesion area in each genotype. Note that Panx1 KO EAE spinal cords exhibit significantly less infiltrating cells than spinal cords from WT mice. Five Panx1 WT and 5 Panx1 KO mice were used for histology; three spinal cord sections from sacral to thoracic areas were analyzed from each mouse. (**C**) Hematoxylin & eosin stained sections of spinal cord from Panx1 WT and Panx1 KO mice obtained at the chronic phase of EAE (35 dpi). Bar histograms on the right represent the mean ± s.e.m values of the percent lesion area in each genotype. At this stage of disease, Panx1 WT and Panx1 KO spinal cords exhibit similar extent of lesion areas. Five Panx1 WT and 5 Panx1 KO mice were used for histology. Three sections from sacral to thoracic spinal cords were analyzed from each mouse. Quantification of lesions is presented as percent area of ventral funiculus white matter occupied by inflammatory cells. P values were obtained using unpaired t-test.

To more directly evaluate the contribution of Panx1 channels to EAE, we immunized Panx1 KO female mice and compared the clinical score with those obtained from Panx1 WT mice. We performed three independent experiments in which mice were followed for 34 days and conducted another additional experiment which was terminated at 13 dpi to harvest tissue. The clinical course of EAE in Panx1 WT and Panx1 KO female mice obtained from four independent experiments is summarized in Figure 2A. Onset of clinical signs was significantly delayed in mice lacking Panx1 (Figs. 2A, B), such that Panx1 WT females developed tail weakness (clinical score 1) at 10.8±0.39 days post immunization (dpi), whereas Panx1 KO females developed tail weakness at 13.3±0.42 dpi (P<0.0001, unpaired t-test; Fig. 2B). Despite the increased latency period, Panx1 KO females fully developed EAE disease by day 14 post-immunization and thereafter clinical scores were similar to those of Panx1 WT females (Fig. 2A). However, during the course of EAE (chronic phase), reduced mortality in Panx1 KO compared to that of Panx1 WT mice was also noted [WT: 35.3% (6 of 17 mice), KO: 11.8% (2 of 17 mice)].

To verify whether the differences in disease outcome seen during the chronic phase of EAE in Panx1 WT mice treated with the Panx1 channel blocker (Fig. 1D) and those of Panx1 KO mice (Fig. 2A) were related to pleiotropic action of MFQ, we compared the neurological scores of untreated and MFQ-treated Panx1 KO mice. Interestingly, in contrast to this possibility, daily administration of MFQ (5 mg/kg; starting 7 dpi) to Panx1 KO mice did not significantly (P = 0.22; N = 10 animals per group) alter disease onset or the neurological scores compared to MFQ-untreated Panx1 KO mice (data not shown).

Our data showing a significant delay in EAE onset in the Panx1 deficient mice suggested that during the initial phase of the disease infiltration of inflammatory cells could be attenuated in these mice. To verify this possibility, histological analysis of inflammatory lesions was performed using H & E stained paraffin sections of spinal cords, focusing on the ventral funiculus white matter, a common site of EAE lesions. Consistent with the hypothesis, at the initial phase of EAE (12–13 dpi), the calculated percent area of ventral funiculus white matter occupied by inflammatory cells in Panx1 WT mice was more extensive (9.6±2.4%, N = 5 mice) than that measured for Panx1 KO mice (3.9±0.9%, N = 5 mice; p = 0.04, t-test) (Fig. 2B). In contrast, at the later phase (35–36 dpi) of disease when no significant clinical difference between the two genotypes was measured, the extent of lesions in the spinal cords of Panx1 WT and Panx1 KO mice was similar (Fig. 2C). No inflammation was observed in non-EAE mice. Thus, these data indicate that lack of Panx1 restricts the extent of inflammation during the initial phase of EAE disease and does not contribute to EAE progression but reduces the incidence of animal death.

### Reduced IL-1β Release from Panx1 KO Splenic Macrophages

**Figure 3:**
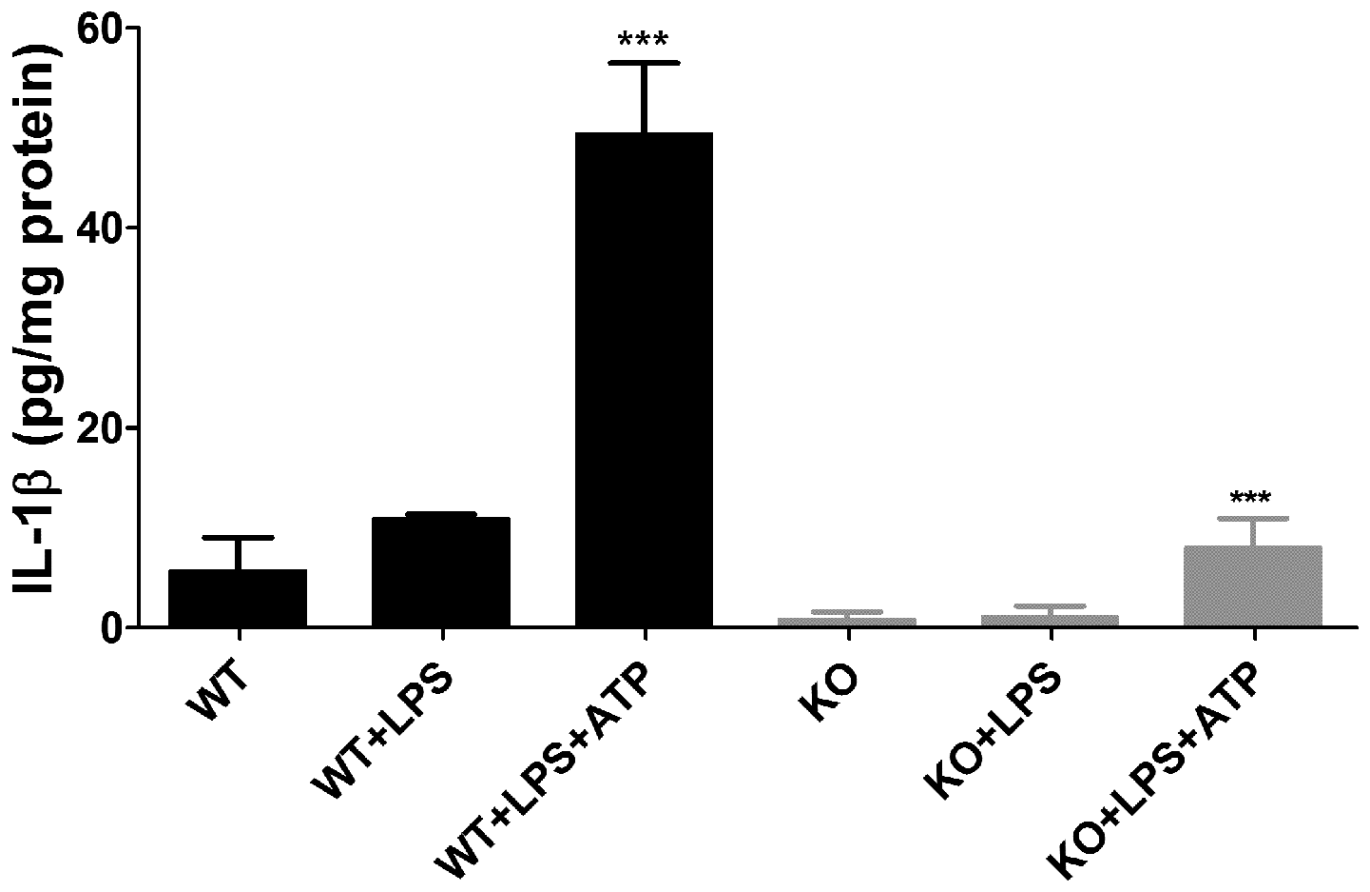
Deficient IL-1β release from activated Panx1 KO splenic macrophages. Bar histograms showing the mean ± s.e.m. values of interleukin-1β (IL-1β) measured from media bathing Panx1 wild type (WT) and Panx1 knockout (KO) macrophage cultures that were untreated, treated with lipopolysaccharide (LPS) and treated with LPS and stimulated with 5 mM ATP. Samples were obtained from 9 mice pooled into three groups and ELISA run in triplicates. ***P<0.001 (ANOVA followed by Newman-Keuls multiple comparison test).

IL-1β released from monocyte-lineage cells contributes to the development of EAE lesions. To evaluate the possibility that the delayed onset of EAE seen in Panx1 KO mice was related to attenuated cytokine production by macrophages, we measured the levels of IL-1β released from activated cultures of splenic macrophages. For that, CD11b-positive adherent splenocytes derived from Panx1 WT and Panx1 KO mice were treated with 1 µg/ml LPS overnight to up-regulate inflammasome components, followed by 5 mM ATP stimulation for 20 minutes and supernatants assayed for IL-1β by ELISA. As shown in Figure 3, LPS activated and ATP stimulated Panx1 WT splenic macrophages released significantly higher IL-1β amounts (49.41±7.1 pg/mg protein) than did activated Panx1 KO cells (8.0±2.9 pg/mg protein). Thus, these results suggest that deficient cytokine production by Panx1 KO splenic macrophages might be related to the reduced area of inflammation in white matter spinal cords which likely delayed the onset of EAE seen in these mice.

### Reduced ATP Release from Spinal Cords of EAE Panx1 KO Mice

**Figure 4:**
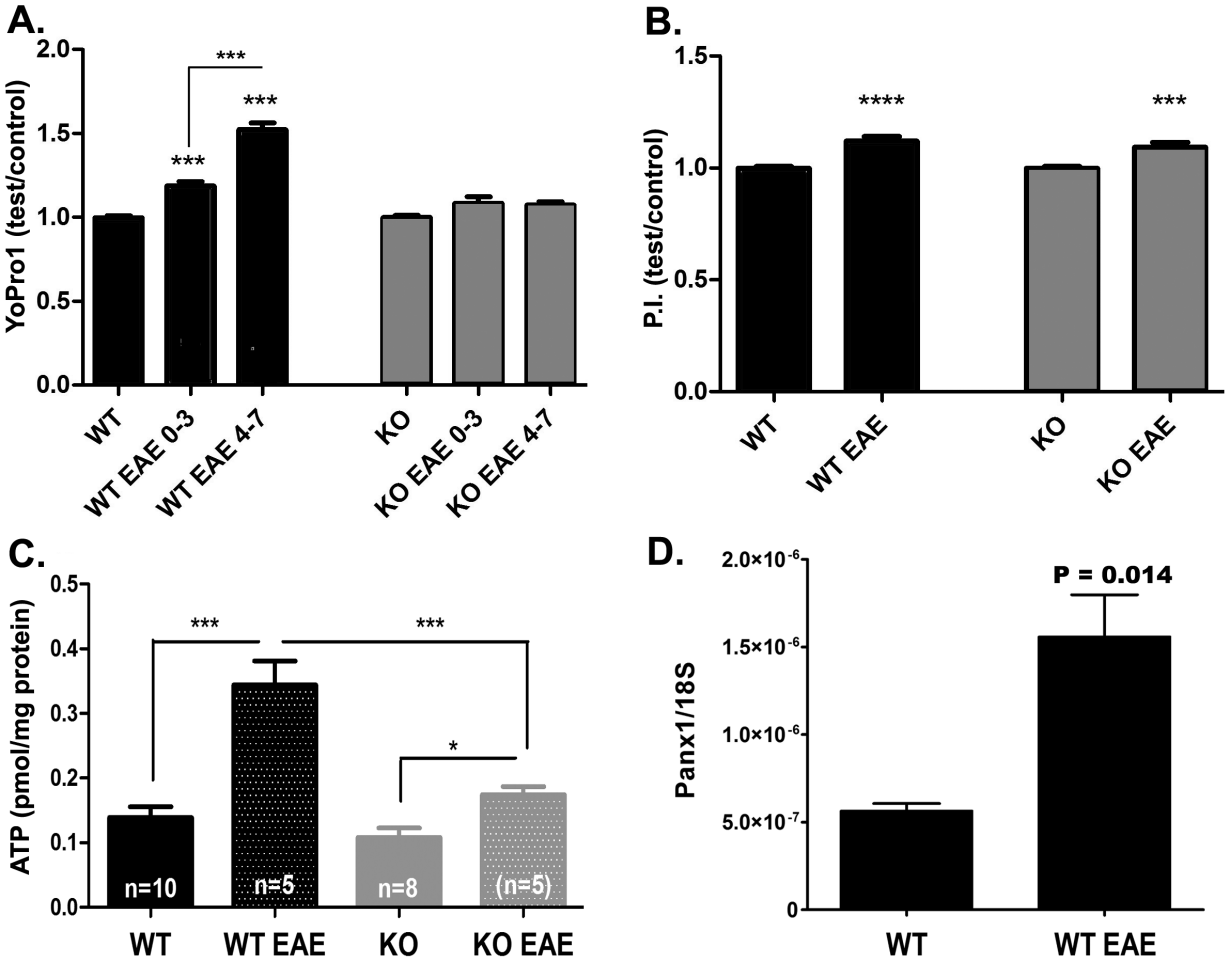
Increased Panx1 channel activity in EAE. (**A**) Bar histograms of the mean ± s.e.m. values of the relative intensity of YoPro uptake (test/control) recorded from Panx1 wild type (WT) and Panx1 knockout (KO) spinal cord slices of control and EAE mice. **P<0.005, ***P<0.001 (ANOVA followed by Newman-Keuls multiple comparison test). Nine Panx1 WT non-EAE, 9 Panx1 WT EAE, 8 Panx1 KO non-EAE, and 13 Panx1 KO EAE mice were used, with a minimum of 167 slices examined per group. Samples from EAE mice were then separated into mildly affected (flaccid tail, hind-limb weakness, clinical score range 0–3) and severely affected (hind-limb paralysis with possible trunk and forelimb involvement, clinical score range 4–7). (**B**) Bar histograms of the mean ± s.e.m. values of the relative intensity of propidium iodide (P.I.) uptake (test/control) recorded from Panx1 wild type (WT) and Panx1 knockout (KO) spinal cord slices of control and EAE mice. ***P<0.001, (ANOVA followed by Newman-Keuls post hoc comparison). Seven Panx1 WT non-EAE, 5 Panx1 WT EAE, 5 Panx1 KO non-EAE, and 7 Panx1 KO EAE mice were used, with a minimum of 71 slices per group examined. (**C**) Bar histograms of the mean ± s.e.m. values of ATP (pmol/mg protein) recorded from ACSF bathing Panx1 wild-type (WT) and Panx1 knockout (KO) spinal cord slices of control and EAE mice. In parentheses are the numbers of animals used. (**D**) Bar histograms of the mean ± s.e.m. values of Panx1 transcript (normalized to ribosomal 18S) measured from RNA samples extracted from cerebellum of naïve and EAE Panx1 WT mice. Samples are from 3 naïve and 3 EAE mice. ***P<0.001 and *P<0.05 (ANOVA followed by Newman-Keuls multiple comparison test); P value in part **D** was obtained using unpaired t-test analysis.

Activation of Panx1 channels can contribute to elevated levels of extracellular ATP, which has excitotoxic effects [3]. To evaluate whether Panx1 channel activity was altered during EAE, we used the dye-uptake assay. For that, we measured the fluorescence intensity of the dye YoPro in the grey matter of acutely isolated slices of cervical spinal cord from Panx1 WT control mice and from mice displaying mild and severe EAE clinical scores. As shown in Figure 4A, YoPro uptake was significantly increased in Panx1 WT mice with EAE as compared to Panx1 WT controls. A dose-dependent increase in dye uptake with disease severity was also recorded. Spinal cord slices from Panx1 WT mice with mild clinical signs (hindlimb weakness) displayed 1.18±0.03 fold higher YoPro uptake compared with control Panx1 WT non-EAE mice (P<0.001). Spinal cords of Panx1 WT mice with severe clinical signs (hindlimb paralysis) displayed significantly higher uptake of YoPro than slices from Panx1 WT mice with mild EAE (p<0.001), and 1.52±0.04 fold higher than control Panx1 WT non-EAE. No significant dye uptake was recorded from spinal cords of Panx1 KO mice with or without EAE (Fig. 4A).

The extent to which increased YoPro fluorescence measured in spinal cords of EAE mice was related to cell damage/death was evaluated using the propidium iodide (PI) uptake assay, which at low concentrations yields high fluorescent signals only in damaged cells. For that, spinal cord slices from EAE and non-EAE Panx1 WT and Panx1 KO mice were bathed in ACSF containing 10 µM PI, and fluorescence intensity measured. Slices from Panx1 WT and Panx1 KO animals sensitized for EAE exhibited a similar 1.12±0.02 fold increase in PI fluorescence intensity, which was significantly higher compared to their non-sensitized controls (P<0.0001; Fig. 4B). Thus, these data suggest that cell damage is likely to account for about 12% of the YoPro uptake measured in spinal cords of EAE mice, such that Panx1-mediated dye-uptake would be expected to correspond to 40% increase in YoPro fluorescence intensity in cases of severe EAE symptoms.

Based on our findings of increased Panx1 activity in EAE, we then quantified the levels of extracellular ATP in spinal cord of these mice. For that, we measured ATP concentration in aliquots of ACSF bathing the spinal cord slices from control Panx1 WT mice and from Panx1 WT mice sensitized for EAE. We found significantly higher levels of ATP released from slices of EAE (0.34±0.04 pmol/mg protein, N = 5 animals; P<0.001) compared to non-EAE (0.14±0.02 pmol/mg protein, N = 10 animals) tissues (Fig. 4C). As expected, spinal cord slices from Panx1 WT mice with EAE released more ATP than those from Panx1 KO mice with EAE (WT: 0.34±0.04 pmol/mg protein, N = 5 mice; KO: 0.17±0.01 pmol/mg protein, N = 8 mice; P<0.001). Spinal cord slices from Panx1 KO mice with EAE displayed a significantly higher level of extracellular ATP (0.17±0.01 pmol/mg protein, N = 5 mice; P<0.05) than non-EAE Panx1 KO mice (0.11±0.01 pmol/mg protein, N = 8 mice; Fig. 4C), indicating that about 0.06 pmol ATP/mg protein is released via a Panx1-independent mechanism, most likely due to cell damage.

Quantitative real time PCR (qPCR) performed on RNA extracted from cerebellum of Panx1 WT mice with EAE (35 dpi; hindlimb paralysis) indicated that Panx1 transcript was 2.8 fold higher than in non-EAE Panx1 WT mice (Fig. 4D).

Together, the dye-uptake and ATP release data indicate that in EAE there is increased Panx1 activity which results in elevated extracellular levels of ATP in spinal cord slices of EAE mice. This increased activity is likely resultant from increased Panx1 expression levels in EAE.

### Increased Expression of P2X_7_ Receptors in EAE

**Figure 5:**
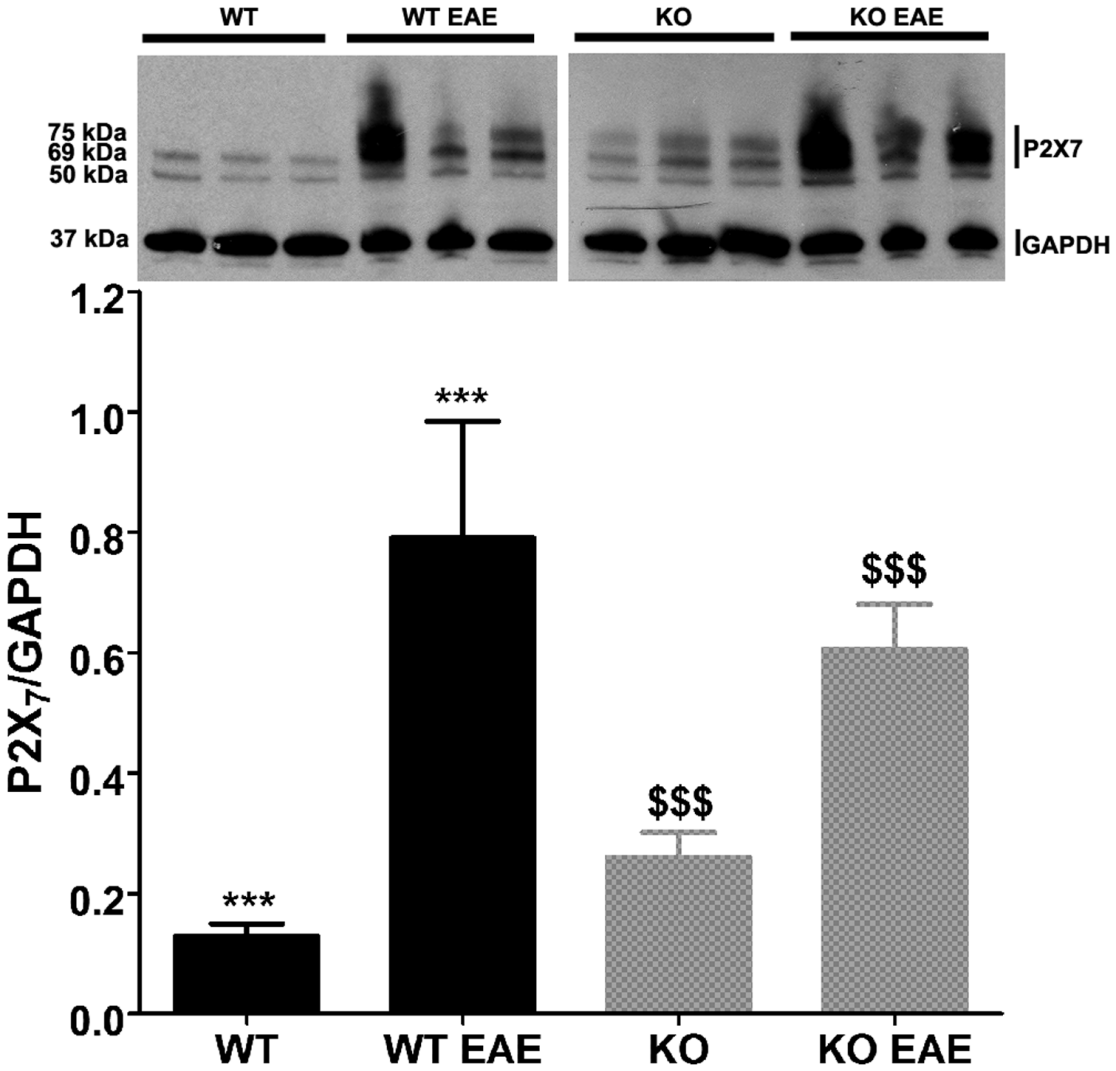
Increased P2X_7_ receptor expression levels in spinal cords of mice with EAE. (**Top**) Western blot showing bands corresponding to the P2X_7_ receptor (69 and 75 kDa) and to GAPDH (37 kDa). Band at 50 kDa likely corresponds to nonspecific staining due to the simultaneous use of two antibodies. (**Bottom**) Bar histograms of the mean ± s.e.m values of P2X_7_R/GAPDH obtained from western blots showing the increased P2X_7_R expression in Panx1 WT and Panx1 KO mice with EAE compared to naïve animals. (***; &&&) P<0.01; t-test.

In view of the results obtained showing that Panx1 KO mice developed as severe EAE as Panx1 WT mice despite decreased extracellular ATP levels, we evaluated whether altered expression levels of the ATP sensitive P2X_7_ receptors could account for these results. Indeed we found that these receptors where expressed at higher levels in lumbar spinal cords from EAE mice of both genotypes, compared to non-EAE mice (Fig. 5). Thus, it is likely that upregulation of P2X_7_R provides a mechanism that could lead to the development of EAE despite reduced ATP levels, as seen the Panx1 KO mice.

## Discussion

Although the initial event in MS is thought to be autoimmune demyelination, axonal damage and tissue atrophy are frequently observed in chronic MS and correlate well with neurological impairment [17]. Likewise, in the chronic phase of murine EAE, a CD4+T cell inflammatory response is frequently accompanied by damage to axons and loss of neurons and oligodendrocytes [18]–[20]. This neurodegenerative phase may be mediated by abundant free radicals, proinflammatory cytokines such as IL-1β and TNFα, glutamate and ATP. In this study, we present evidence that Panx1 channels contribute to EAE pathology, in part by releasing ATP from diseased tissue. A recent report also indicates that mice with global deletion of Panx1, or with neuronal-targeted conditional deletion of Panx1, are protected from excitotoxic cell death in retina ischemia reperfusion, consistent with a role for Panx1 in direct neurotoxicity [14].

In the present study, we found that female Panx1 KO mice exhibited a significant 2.5-day delay in the onset of clinical signs, whereas at 33 dpi the Panx1 KO mice were as sick as the surviving Panx1 WT mice. We also recorded decreased number of deaths among Panx1 KO than Panx1 WT mice with EAE. Consistent with their less severe clinical scores in the acute phase, histopathologic analysis of EAE spinal cords at 12–13 dpi showed that inflammatory lesions were decreased in the Panx1 KO. Parenchymal lesions were still present in the Panx1 KO EAE spinal cord, but were less frequent and tended to be smaller in size than those found in Panx1 WT EAE lesions, suggesting that Panx1 is not strictly required for leukocyte blood brain barrier transmigration and lesion initiation, but does participate in lesion expansion.

We also showed that MFQ, a Panx1 channel blocker, delayed and provided protection against clinical signs of EAE in rats and mice. It remains unclear why Panx1 WT mice treated with MFQ, but not Panx1 KO mice, showed decreased disease severity in the chronic phase of EAE. One possibility is related to the hypomorphic phenotype of Panx1 KO mice which display about 30% Panx1 mRNA of that of WT mice [21]. Another possibility may be due to pleiotropic effects of the drug. For instance, mefloquine has been shown to inhibit p-glycoprotein transporter [22] and the adenosine A2A receptor [23], two important players in EAE [24], [25]. However, given that MFQ did not alter the outcome of EAE when administered to Panx1 KO mice, these possibilities seem unlikely. Nonetheless, we show by pharmacologic inhibition and genetic deletion that in both conditions Panx1 contributes to disease expression, and in the latter case, also increases mortality of mice with EAE.

In this study we also examined whether Panx1 channels were activated in EAE by measuring the amount of ATP released and YoPro uptake in acute spinal cord slices from mice sensitized for EAE and healthy non-EAE control mice. In control conditions we found that these channels are likely quiescent since equivalent amounts of ATP were detected in the ACSF bathing spinal cord slices of healthy Panx1 WT and Panx1 KO mice. Similarly, no significant differences in dye-uptake were detected in these tissues. In the EAE setting, however, we found that Panx1 channels are active and contribute to ATP release from CNS tissues given that Panx1 KO EAE spinal cords released significantly less ATP than did Panx1 WT EAE spinal cords. It is also likely that the increased Panx1 mRNA expression levels detected in chronic EAE tissues of Panx1 WT is related to the increased levels of extracellular ATP detected in these tissues. At present, it is difficult to evaluate by western blot whether Panx1 expression is altered and/or whether there are post-translational changes during EAE that affect Panx1 activity. Recent study indicates that different Panx1 antibodies yield different bands of sizes in different tissues, including Panx1 knockout mice [26]. Our findings are similar to those previously reported for retinal tissues, where, under normoxic conditions, Panx1 WT and Panx1 KO tissues and cellular permeability to dye were found to be similar, changing only after 30 min of oxygen-glucose deprivation [14].

There are several possible ways by which Panx1 channels might contribute to EAE. One mechanism is by inducing ATP-dependent excitotoxicity, due to the extrusion of ATP through open Panx1 channels. High extracellular ATP levels activating P2X_7_ receptors, either alone or in a physical association with Panx1 in the membrane, would result in membrane permeabilization, influx of Ca^2+^, and cell death. Indeed, previous work has shown that exogenous application of ATP leads to demyelination and oligodendrocyte losses, and that such effects could be prevented by pharmacologic inhibition of the ATP-sensitive P2X_7_R [4]. Our experiments using *ex-vivo* spinal cords indicate that Panx1 is a predominant mechanism for ATP release in EAE, but we cannot distinguish the cellular source. Panx1 protein and transcript are expressed in neurons, astrocytes, oligodendrocytes, macrophages, and T cells [3], [8], [9], [14], [27]–[30]. Because our ATP experiments were conducted on spinal cords during the chronic phase, when the T cell burden is low (Fig. 2), this suggests that at least in the latter phase of the disease, Panx1-mediated ATP release does not come from infiltrating lymphocytes. The fact that P2X_7_R was found to be upregulated in chronic EAE spinal cords, suggest the presence of a mechanism by which disease progresses despite reduced ATP release. This possibility could explain the lack of clinical score improvement in Panx1 KO mice compared to those of surviving WT mice; nevertheless, our results suggest a certain degree of protection in the absence of Panx1 given that number of EAE related death in the null genotype was reduced compared to that of Panx1 WT mice.

The contribution of Panx1 to EAE can also be due to increased levels of adenosine, which is rapidly generated from ATP by CD73. Increased adenosine levels are observed after cellular stress, neuroinflammation and brain injury (for reviews see, [31]–[33]). However, it is not clear whether adenosine has protective or deleterious roles in multiple sclerosis models. Mice that lack CD73 expression have been shown to be protected from EAE [34], and blockade of adenosine receptors with caffeine, a broad spectrum adenosine receptor antagonist have been shown to attenuate EAE symptoms [35]. However, reports in which the deletion of specific adenosine receptors was evaluated indicate a dependence of disease severity on receptor subtypes. Thus, A1 and A2A adenosine receptor activation is protective in EAE models ([24], [36] but see [34]) whereas the opposite effect is observed with A2B receptors [37].

Another way by which Panx1 may contribute to EAE involves inflammasome activation. In neurons and astrocytes, Panx1 and P2X_7_ receptors co-immunoprecipitate with each other and with components of the inflammasome [8]. In this modality, ATP released through Panx1 activates P2X_7_R triggering the recruitment of caspase-1 to the inflammasome, which then cleaves pro-IL1β into its mature form [30], [38]. IL-1β is found at high levels in EAE and MS lesions [39], primarily derived from macrophages and microglia, but also can be produced in neurons and astrocytes. IL-1β is a potent activator of astrocytes [40], and is important for inducing IL-17 production from CD4 and gamma-delta T-cells [41]–[43]. Disruption of the Panx1-P2X_7_R complex, in the absence of Panx1 signaling, with consequent prevention of inflammasome activation and IL-1β processing represents an attractive explanation for the delayed onset of clinical and pathological signs of EAE observed in our Panx1 KO mice and in rats treated with MFQ. Such possibility is consistent with our results showing that IL-1β secretion is impaired in ATP-stimulated splenic macrophages from Panx1 KO mice. Similar findings were also reported for macrophages treated with Panx1 siRNA [6], and for neurons and astrocytes treated with the Panx1 inhibitor probenecid [8]. Moreover, retinas from global and neuronal-targeted Panx1 KO were also shown to fail to activate the inflammasome and the processing of IL-1β triggered by ischemic events [14].

Several lines of evidence also point to a role for P2X_7_R itself in white matter damage. P2X_7_R expression is elevated in MS white matter [4], [44]. Similarly, P2X_7_R is elevated in neurons and astrocytes during acute EAE and after symptom recovery [45], [46]. A gain-of function P2X_7_R polymorphism that increases Ca^2+^ permeability occurs at higher frequency in MS patients than in the general population [47]. The contribution of P2X_7_R signaling to neuroinflammation has also been directly tested in EAE. Genetic deletion of P2X_7_R alters severity of and susceptibility to EAE, although differing effects were found in independently derived P2X_7_R KO mice [48], [49]. The P2X_7_R inhibitors brilliant blue G (BBG) and oxidized-ATP (oATP) attenuate EAE signs, even when administered after onset of disease [4]. Interestingly, it has been recently shown that both BBG and oATP are potent inhibitors of Pannexin1 channels [50], [51].

It should be kept in mind, however, that mechanisms other than the ones highlighted here are likely involved in EAE. Also possible is that responses to the absence of Panx1 vary between the immune system and the CNS. Thus, the lack of Panx1 may attenuate the immune response during the initial stages of EAE (as supported by data displayed in Fig. 3), while ongoing CNS damage in the chronic EAE phase may rely, in addition to Panx1, in other yet unknown mechanisms**.** Micro-array analysis would certainly provide an unbiased approach to unveil such mechanisms.

In summary, we have shown that heightened ATP release is characteristic of the EAE spinal cord, and that most of the ATP release can be accounted for by Panx1 channel activity. Panx1 is shown to contribute to both early and late stages of EAE, but the disease-inducing effect of Panx1 seems most profound during the acute phase. Genetic ablation or blockade of Panx1 delays the onset of clinical signs of EAE. During the chronic phase, wild-type mice had high incidence of mortality, while Panx1 KO mice were protected from this mortality, although surviving mice expressed clinical signs of disease. Our data suggest that a Panx1-mediated pathway (ATP release and/or inflammasome activation) contributes to neuroinflammatory damage in the diseased spinal cord, and open the possibility that modulation of Panx1 could be a novel therapeutic target in multiple sclerosis.
