# Correction: Contribution of Pannexin1 to Experimental Autoimmune Encephalomyelitis

**DOI:** 10.1371/annotation/cedbee08-9c0e-42e3-862f-df7409c273ef

**Published:** 2013-12-16

**Authors:** Sarah E. Lutz, Estibaliz González-Fernández, Juan Carlos Chara Ventura, Alberto Pérez-Samartín, Leonid Tarassishin, Hiromitsu Negoro, Naman K. Patel, Sylvia O. Suadicani, Sunhee C. Lee, Carlos Matute, Eliana Scemes

The correct address for Prof. Carlos Matute is: Achucarro Basque Center for Neuroscience, CIBERNED and Departamento de Neurociencias, Universidad del Paıs Vasco, Leioa, Spain.

There was an error in the last sentence of the eighth paragraph of the Discussion section. The corrected sentence should read: “Interestingly, it has been recently shown that BBG but not oATP is a potent inhibitor of Pannexin1 channels [50,51].”

Reference [51] should be updated to: Wang J, Jackson DG, Dahl G (2013) The food dye FD&C Blue No. 1 is a selective inhibitor of the ATP release channel Panx1. J Gen Physiol 141(5):649-656. 

